# A straightforward approach for gated STED-FCS to investigate lipid membrane dynamics

**DOI:** 10.1016/j.ymeth.2015.06.017

**Published:** 2015-10-15

**Authors:** Mathias P. Clausen, Erdinc Sezgin, Jorge Bernardino de la Serna, Dominic Waithe, B. Christoffer Lagerholm, Christian Eggeling

**Affiliations:** a MRC Human Immunology Unit, Weatherall Institute of Molecular Medicine, University of Oxford, Headley Way, Oxford OX3 9DS, United Kingdom; b Wolfson Imaging Centre, Weatherall Institute of Molecular Medicine, University of Oxford, Headley Way, Oxford OX3 9DS, United Kingdom

**Keywords:** Plasma membrane dynamics, Super-resolution STED microscopy, Fluorescence correlation spectroscopy, Lipid diffusion, Time-gated single-photon counting

## Abstract

Recent years have seen the development of multiple technologies to investigate, with great spatial and temporal resolution, the dynamics of lipids in cellular and model membranes. One of these approaches is the combination of far-field super-resolution stimulated-emission-depletion (STED) microscopy with fluorescence correlation spectroscopy (FCS). STED-FCS combines the diffraction-unlimited spatial resolution of STED microscopy with the statistical accuracy of FCS to determine sub-millisecond-fast molecular dynamics with single-molecule sensitivity. A unique advantage of STED-FCS is that the observation spot for the FCS data recordings can be tuned to sub-diffraction scales, i.e. <200 nm in diameter, in a gradual manner to investigate fast diffusion of membrane-incorporated labelled entities. Unfortunately, so far the STED-FCS technology has mostly been applied on a few custom-built setups optimised for far-red fluorescent emitters. Here, we summarise the basics of the STED-FCS technology and highlight how it can give novel details into molecular diffusion modes. Most importantly, we present a straightforward way for performing STED-FCS measurements on an unmodified turnkey commercial system using a time-gated detection scheme. Further, we have evaluated the STED-FCS performance of different commonly used green emitting fluorescent dyes applying freely available, custom-written analysis software.

## 1 Introduction

The plasma membrane is a dynamic environment that plays host to a variety of interacting molecular species. The functional behaviour of the constituent species is mediated through a variety of protein–protein or protein–lipid interactions, some of which are supported by membrane-associated structures such as the cortical cytoskeleton, and all of which restrict the free lateral movement of the constituent species [1], [2], [3], [4]. These interactions are often short-lived, and in addition, many of the involved molecules are very mobile. Consequently, the direct imaging of such molecular interactions using optical microscopy is challenging. For example, the acquisition time of scanning confocal microscopy is usually too slow to follow dynamics such as the formation and disassembly of transient molecular clusters. Further, to visualise molecular assemblies, molecules have to be unevenly distributed between an un-clustered and clustered state on a spatial scale that can be resolved by optical microscopy [4], [5]. Many experiments are therefore performed on a specific state of a cell at a fixed time point by use of a variety of chemical cross-linking methods. Such procedure will however not allow the direct observation of transient states. In addition, it may result in artefacts, i.e. some membrane molecules are still mobile even after fixation [6], [7].

Several optical techniques have emerged over the last decades, which bring along the required temporal resolution to investigate fast molecular diffusion and to highlight transient interactions in molecular assemblies [2], [8], [9], one of which is fluorescence correlation spectroscopy (FCS) [10], [11], [12]. In FCS, the temporal fluctuations of the observed fluorescence signal are monitored over time as labelled molecules diffuse in and out of an observation area or volume, and the auto-correlation function of these fluctuations is calculated. The decay time of this correlation function is proportional to the average transit time of the molecules moving through the observation area. Hindrances or anomalies in diffusion therefore result in a shift of the correlation curve towards larger times and the stretching of the decay.

FCS is usually applied on a confocal microscope, allowing access to fluctuating fluorescence intensity time traces with single-molecule sensitivity [13]. The use of focused light on such lens-based (or far-field) microscope allows the investigation of cellular structures without an optical element touching the sample, reducing the invasiveness of the observation. But the non-invasiveness comes at a price that the light, because of diffraction, cannot be focused to a dimension smaller than approximately 200 nm for visible light. This places an effective lower limit on the size of the observation spot and thus the spatial resolution of a conventional far-field microscope [14]. Consequently, FCS experiments on a far-field microscope typically cannot resolve nanoscopic hindrances in the diffusion and may miss important details on anomalous diffusion due to e.g. transient interactions. For example, FCS at diffraction-limited resolution cannot distinguish between a reduction in free Brownian diffusion and transient halts in diffusion that have a duration that is equivalent or less than the transit time through the observation spot [4], [5], [15]. This issue has been addressed by different variations of diffraction-limited FCS (z-scan FCS, sampling-volume-controlled (SVC)-FCS, and spot-variation (sv)FCS) [3], [16], [17], [18], [19] by using correlation data recorded for a range of observation area sizes. The resulting dependency of the average transit time through the observation spot allows for gaining more detailed information of (anomalous) diffusion modes. Using svFCS in 200 nm to >1 μm large observation areas, the diffusion characteristics of plasma membrane molecules could be assigned to different diffusion modes [20]. However, this assignment is only realised by an extrapolation to even smaller areas, and further details of the molecular dynamics such as molecular interaction times or cluster areas can only be estimated. A remedy to all these limitations is the recording of FCS measurements with observation areas <200 nm [21], [22]. This has been facilitated by recording FCS data near nanometer-sized apertures, as for example realised by placing the membrane sample in zero-mode waveguides [23], or on a pattern of isolated nano-apertures milled in a metallic film [24], [25], [26], or by positioning a small tip with a nanometer-sized aperture near the sample (near-field microscopy) [27]. Unfortunately, in all cases the sample has to be brought into nanometer-proximity to a surface. This might introduce unforeseeable bias, and further will preclude studies of intra-cellular dynamics, which is possible with far-field optics.

Researchers tried to overcome the resolution limit of lens-based optical microscopy for decades. However, significant progress towards breaking the diffraction limit was not made until the 1990s when the key idea was formulated that would allow far-field fluorescence microscopy to be performed without any limitation in spatial resolution [28]. The first implementation of such an optical nanoscope (or super-resolution microscope), Stimulated Emission Depletion (STED) microscopy [28], [29], introduced the idea of implementing an optically driven reversible transfer between states of different emission properties of the fluorescence labels, such as a dark and a bright state, thereby allowing the modulation or reversible inhibition of fluorescence emission in space and time [30], [31], [32], [33]. In a STED microscope, the area in which fluorescence emission is allowed (i.e. the observation spot) is tuned to sub-diffraction scales by the addition of a STED laser forcing the fluorescent labels to their dark ground state in the focal periphery using stimulated emission. It has been shown that FCS observations in such reduced observation spots can overcome aforementioned limitations of conventional FCS recordings that are diffraction limited [4], [5], [15], [22], [34], [35], [36], [37], [38], [39], [40], [41], [42]. Specifically, the ability to tune the size of the observation spot by the intensity of the added STED laser introduces a precise way of differentiating free from anomalous diffusion, similar to svFCS [17], but now by directly measuring at the relevant nanometer scale.

STED-FCS recordings have thus far only been recorded in a few laboratories, mainly due to the greater care in optical alignment and larger complexity of the STED microscope setup for STED-FCS recordings compared to pure imaging. One reason is the large signal-to-background ratio that has to be accomplished to realise distinct fluctuations in the fluorescence signal (due to single-molecule transits) that are required for accurate FCS data recordings [35], [36], [43], [44]. The implementation of the STED-FCS technology on a turnkey commercial instrument would push its more wide-spread use. Previous realisations of STED-FCS on a commercial microscope were still hampered by significant alteration to the system and/or rather long acquisition times [39], [40].

Here, we demonstrate the straightforward use of STED-FCS on an unmodified, turnkey commercial STED setup, opening up ways for a more wide-spread use of the STED-FCS technology. We apply the technique in combination with a freely available, custom-written analysis software to evaluate the performance of multiple green-emitting fluorescent lipid analogues diffusing in supported lipid bilayers (SLBs). We further show how a variant of the STED-FCS technique using a gated detection scheme allows us to generate FCS data for different sizes of the observation spot from a single measurement, thus reducing the total measurement time.

## 2 Material and methods

### 2.1 Lipid analogues

We tested different fluorescent phosphoethanolamine (PE) lipid analogues labelled with the organic dyes Oregon Green 488 (Life Technologies Ltd, Paisley, UK), TopFluor (Avanti Polar Lipids, Alabaster, Alabama, USA), Bodipy FL (Life Technologies), Atto488 (Atto-Tec GmbH, Siegen, Germany) and Abberior Star 512 (Abberior GmbH, Goettingen, Germany). The organic dyes were coupled to either the head group (Atto488, Abberior STAR 512, Oregon Green 488) or the acyl chain (Bodipy FL, TopFluor) of the phospholipid dipalmitoyl phosphatidylethanolamine (DPPE; TopFluor, Atto488, Abberior Star 512) or dihexadecFanoyl phosphatidylethanolamine (DHPE; Oregon Green 488, Bodipy FL), as provided by the respective companies. We refer to all the phospholipids as PE throughout the text.

### 2.2 Supported lipid bilayer preparation – spin coating

We measured lipid diffusion by STED-FCS in simple one-component fluid-phase supported lipid bilayers (SLBs). Such SLBs are commonly used to calibrate the resolution in STED-FCS measurements as the lipids in this system undergo free Brownian motion. The SLBs can be prepared in different ways such as by vesicle fusion or spin coating. Here, we used the spin coating technique with some adaptation from the original protocol [45]. SLBs were made from a lipid stock solution with 1 mg/mL DOPC (1,2-dioleoyl-sn-glycero-3-phosphocholine, Avanti Polar Lipids) and the fluorescent lipid analogue of interest dissolved in Chloroform:MeOH (2:1 v:v) (Sigma Aldrich Co Ltd, UK). The ratio of un-labelled to labelled lipids was 5000–20,000:1. The solution was stored at −20 °C. Before each SLB preparation, the lipid stock solution was vortexed vigorously. 20 μL of the lipid solution was then dropped onto a piranha-cleaned standard microscope cover glass (diameter 25 mm, No. 1.5 thickness) placed on top of the spin coater (Chemat Technology) chuck and immediately spun at 3000–4000 rpm for 40 s. This allows for the solvent to evaporate and a lipid film to form. The cover glass was then placed in a microscopy chamber and hydrated with 1 mL buffer solution (150 mM NaCl, 10 mM HEPES (Lonza Biologics plc, Slough, UK)) during which, areas of single bilayers are formed. The SLB was stable for several hours.

### 2.3 STED-FCS microscope

**Fig. 1:**
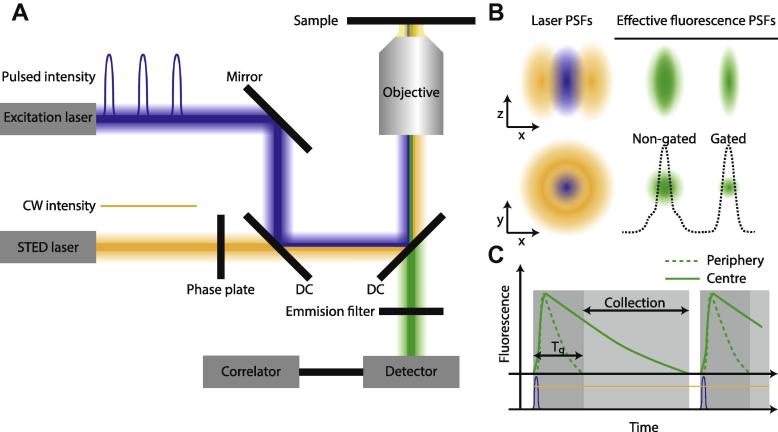
Illustration of gated STED-FCS. (A) Experimental setup with lasers and laser beams (blue: excitation, orange: STED), phase plate, dichroic mirrors (DC), fluorescence beam (green), emission filter, objective lens, fluorescence detector and correlator electronics. The excitation and STED lasers are superimposed using the right combination of DCs. In the presented scheme, the excitation laser has a pulsed modality, while the STED laser is running in CW. (B) Point Spread Functions (PSFs) of the different lasers (left panels); the excitation laser (blue) shows a normal Gaussian profile, while the STED laser (orange) has a doughnut shaped profile in the *x*–*y* plane caused by introducing a phase plate in the beam path. (Right panels) Without gating the effective fluorescence spot has a pedestal due to inefficient depletion shortly after the arrival of the excitation pulse. This pedestal is removed by gating. (C) Fluorescence decay (green) over time following an excitation laser pulse (blue) and under the influence of the continuous action of the doughnut-shaped CW STED laser (orange). The decay at the centre of the focal spot (straight line) is unaffected by the STED laser, and therefore shows a relatively long fluorescence lifetime, while the periphery of the focal spot shows a reduced (but not zero!) fluorescence lifetime decay (dashed line). By only including the signal from after the peripheral fluorescence is depleted (gating with a delay *T_g_* relative to the excitation pulse), and a smaller and Gaussian intensity profile of the effective fluorescence spot is created.

We applied STED-FCS on an unmodified commercial STED setup (SP8x, Leica Microsystems GmbH, Mannheim, Germany). This setup is equipped with a white-light-laser (WLL, NKT Photonics) for flexible choice of excitation wavelengths, and a high-power STED laser at 592 nm. While the WLL system delivers pulse trains of 80 MHz repetition rate and approx. 80 ps pulse width, the STED laser runs in continuous-wave (CW) mode. In addition, the excitation laser light is focused to a diffraction-limited spot, while the STED laser beam is modified in such a way to generate a doughnut-shaped focal intensity distribution featuring a central intensity zero (Fig. 1). The master power of the WLL was set to 30%. Subsequently excitation at 488 nm was used at 1–25% output power (total power of 0–7 μW measured directly at the focal plane). The CW-STED 592 nm laser was operated at 0–100% output power (100% of master power, a total power of 0–212 mW measured directly at the focal plane). Before each series of measurements the auto-alignment procedure (super-imposing the excitation laser and the depletion lasers) was performed. This procedure was repeated every 4 h. A 100x 1.4 NA oil immersion objective realises focusing of the overlaid excitation and STED laser beams as well as collection of the emitted fluorescence. The latter is through the confocal pinhole (in our experiments set to 1 Airy unit) imaged onto a single-photon-counting avalanche photo-diode (APD; Micro Photon Devices, PicoQuant, Berlin, Germany) in the external port of the microscope with a band-pass filter selecting fluorescence specifically between 500–550 nm. The APD signal was recorded with a time-correlated single-photon-counting (TCSPC) detection unit (Picoharp 300, PicoQuant), which was delivered with and is fully integrated into the microscope, and which saves the raw photon stream (for each detected photon the macroscopic time within the total data acquisition, and the microscopic time to the next excitation pulse is recorded). Using the microscope software (SymPhoTime, PicoQuant), this allows reconstruction of fluorescence lifetime decays as well as fast calculation of FCS data [46], [47], specifically from photon subsets if required [48]. The recordings were directly controlled by the Leica LAS AF software, which communicates with the PicoQuant SymPhoTime software as an already integrated FCS package in LAS AF.

### 2.4 STED-FCS measurements

The SLB sample chamber was placed on top of the microscope objective. The optimal z-position of the SLB was chosen by maximising the fluorescence intensity counts, and minimising the correlation amplitude and decay time, all of which ensures that the SLB is in the centre of the observation spot. Once the z-position was optimised, the adaptive focus control was enabled to avoid drift in the z-direction between measurements. The duration of each measurement was 10 s. For each position on the SLB, a full series of power settings (excitation or STED) was performed with three repetitions per power setting. Three different positions were chosen per SLB, and the FCS protocol was repeated for three independent sample preparations.

### 2.5 The gated STED-FCS approach

The combination of pulsed excitation with CW-STED lasers has several advantages compared to the conventional all-pulsed modality. This arrangement avoids temporal alignment of the pulses of the excitation and STED lasers, as well as realises the use of a more compact and less complex laser systems (e.g. laser diode or solid-state laser systems in comparison to Ti:Sa laser-based arrangements, often including an additional optical parametric oscillator (OPO)) [44], [49]. However, the use of a CW-STED laser brings two main drawbacks compared to the all-pulsed STED modality. Firstly, much higher average laser powers have to be applied [50]. This follows as tightly synchronised trains of excitation and STED pulses, as realised in the pulsed modality, yield a more optimised efficiency of stimulated emission compared to the time-averaged action of the CW light. Secondly, the CW-STED modality results in the fact that a non-negligible part of the fluorescence labels spontaneously emit light before having been exposed to enough of the STED light. As a consequence, residual fluorescence outside the zero-intensity point of the CW-STED light leads to a pedestal in the effective observation spot, resulting in somewhat lower-contrast images and Lorentzian rather than a Gaussian intensity profile of the emitted fluorescence from the observation spot (Fig. 1B and C) [43]. Conventional FCS theory is based on Gaussian profiles [13]. Consequently, FCS data recorded with CW-STED lasers deviate from conventional FCS theory due to the anomaly introduced by the Lorentzian profile [49], or else require more sophisticated data fitting routines designed to accommodate this deviation [51], [52].

The problem of the pedestal inherent to the CW-STED modality can be solved by implementing a time-gated detection scheme (Fig. 1B and C) [49], [53]. Besides suppression of general background for an improved single-molecule detection (for example [48], [54]) or of scattered laser light or un-depleted fluorescence signal during the excitation pulse [55], time-gated detection also reduces the aforementioned pedestal in the effective observation spot of the CW-STED modality [49], [53], [56]. As a consequence, FCS data recorded with gated detection are again well described by theory based on Gaussian fluorescence emission profiles [49].

Specifically, in the STED gating process only photons arriving within a time window at a certain delay *T_g_* after the excitation pulse are included for data processing (Fig. 1C). This favours fluorescence emitted at the focal centre, which are not subjected to the STED light and therefore still exhibit a long fluorescence lifetime. In contrast, spontaneous fluorescence of labels with a short fluorescence lifetime, i.e. that experience increasing levels of STED light, is neglected [49], [53] (Fig. 1C). As a result, peripheral fluorescence emission is specifically reduced, which further narrows the resulting effective observation spot (Fig. 1B), however at the expense of signal loss due to the general rejection of signal [56].

### 2.6 STED-FCS analysis

We used the microscope’s software (SymPhoTime, Picoquant) only for quick data inspection, but have custom written an FCS data analysis software to quickly facilitate batch generation and analysis of a large number of gated FCS data. The custom written software is called FoCuS – point, and is freely available at https://github.com/dwaithe/FCS_point_correlator. The raw fluorescence intensity data was loaded into the software and processed using a variant of a fast TCSPC algorithm [57], generating the fluorescence lifetime histogram and correlation curves for each data. Multiple FCS curves with different time gates were calculated from one data file with the time gates chosen by inspection of the lifetime histogram. The correlation curves were fit by the same software, using a model for one component, two-dimensional diffusion with a single triplet state [36],

(1) $$G(t_{c})=G(0)\frac{1}{1+{\left(\frac{t_{c}}{\tau_{D}}\right)}^{\alpha }}\left(1-T+{\mathrm{Te}}^{-\frac{t_{c}}{\tau_{T}}}\right)$$

with correlation function *G*(*t_c_*) and amplitude *G*(0), correlation time *t_c_*, average transit time *τ_D_*, triplet fraction *T*, triplet correlation time *τ_T_*, and anomaly factor *α*. The anomaly factor *α* is introduced to quantify the deviation of the correlation curve from describing free diffusion through an observation spot with a Gaussian intensity profile. It is 1 for a correct description and deviates from 1 for, for example, a more Lorentzian intensity profile. Together with the average total count-rate 〈*F*〉 the amplitude *G*(0) allows for estimating the single-molecule brightness (or counts-per-molecule, cpm)

(2) cpm=G(0)〈F〉

The data presented was fitted in the time window of *t_c_* = 0.001–200 ms. Confocal measurements were used to determine *T*, and triplet correlation time *τ_T_*, which were then fixed during further fitting. The quality of each fit was visually inspected, and fits with an anomaly factor α of 0.8 < *α* < 1.2 were retained, at least in the presented experiments on free diffusing lipids.

## 3 Results

### 3.1 Evaluating fluorophores for STED-FCS performance

For testing the performance of STED-FCS on our unmodified commercial microscope we investigated the free diffusion of fluorescent phosphoethanolamine (PE) lipid analogues labelled with different green-emitting organic dyes (see Section 2.1) in a one-component DOPC supported lipid bilayer (SLB), where the lipids exhibited free diffusion (see Section 2.2). All of these fluorescent PE analogues could be used with a STED laser wavelength of 592 nm. The quality of a fluorescent label used in FCS recordings is primarily determined by its ability to exhibit low photo-bleaching and high single-molecule brightness (or count-rate per molecule, cpm). Photo-bleaching was observed to bias the FCS analysis since fluorescence emission diminishes before molecules have left the observation spot, resulting in an apparent reduction of the transit times and thus a bias towards faster molecular mobility [58]. High single-molecule brightness (cpm), on the other hand, ensures less noisy fluorescence fluctuations and hence higher-quality FCS data [59], [60], [61]. A poor performance with regards to these two aspects in the confocal case will obviously result in less than optimal performance also in the reduced observation spots created by STED [36]. A bias due to photo-bleaching before leaving the observation spot will be higher for the long transits through the large confocal spot relative to the shortened transits of the spots confined by the STED light [36].

**Fig. 2:**
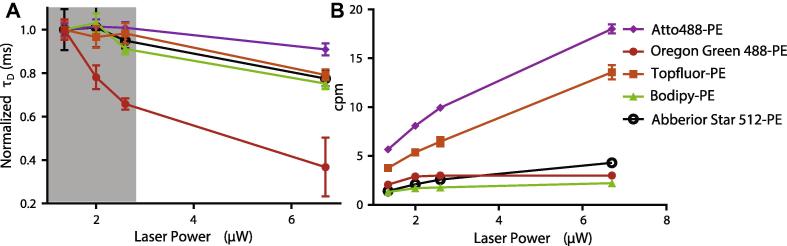
Photo-stability and molecular brightness of different fluorescent PE-lipid analogues diffusing freely in the fluid DOPC SLB, as determined by confocal FCS. (A) Transit times through the observation volume for varying laser power. The grey shaded area shows the region where there is no significant photo-bleaching for all tested analogues but Oregon Green 488-PE. All transit times are normalised to the lowest power used. (B) Count per molecule (cpm) (average count rate divided by number of molecules) for varying laser power. Bars are standard deviations from at least five independent measurements.

Consequently, we first tested the fluorescent lipid analogues for their performance concerning photo-stability and molecular brightness in confocal FCS recordings only. We recorded FCS data for different time-averaged powers *P_exc_* of the 488 nm excitation laser (measured directly at the focal plane), extracted both the average transit time *τ_D_* (Eq. (1)) and the single-molecule brightness cpm (Eq. (2)) from each curve, and then plotted these parameters in dependence of *P_exc_* (Fig. 2). As expected, *τ_D_* decreased for high *P_exc_* due to enhanced photo-bleaching. This effect was most pronounced for the Oregon Green 488 label, where the reduction happened instantly, while it was only noticeable above 3 μW for the other labels (Fig. 2A). The single-molecule brightness was highest for the Atto488 label, while the performance in this respect was much worse for Bodipy and Oregon Green 488 (Fig. 2B). Note that the molecular brightness saturated with increasing laser power due to dark state transitions and photo-bleaching [58]. Consequently, we proceeded only with testing the performance of TopFluor, Atto488 and Abberior Star 512 labelled PE lipid analogues in gated STED-FCS.

### 3.2 Optimising STED-FCS by time gating

**Fig. 3:**
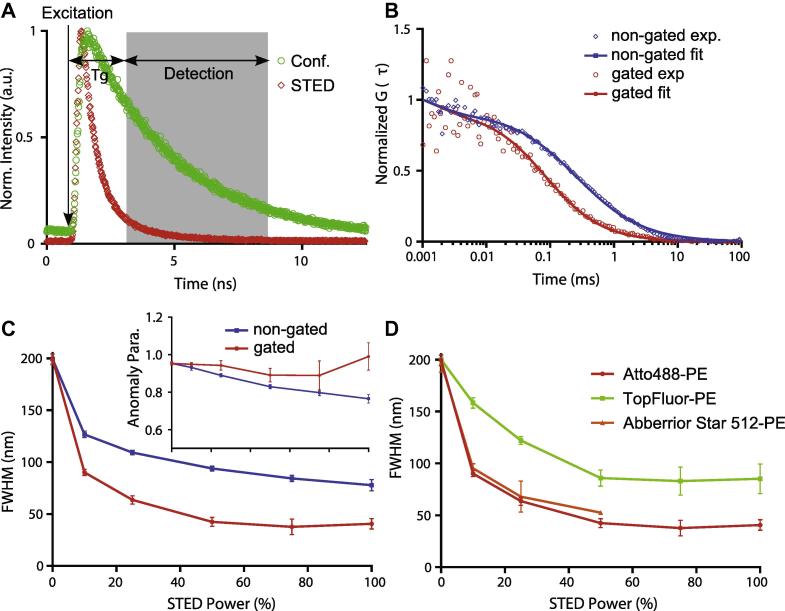
Gated vs. non-gated STED-FCS for selected fluorescent PE lipid analogues diffusing freely in the DOPC SLB. (A) Fluorescence lifetime and gating principle; the plot shows the experimental fluorescence lifetime decay of Atto488-PE in the DOPC SLB for confocal (green) and STED (red) recordings. The reduced lifetime in the case of STED is due to the action of the STED laser in the periphery of the excitation beam, as the depletion is incomplete shortly after the excitation pulse, this region is excluded from the analysis, which only include the grey shaded area. (B) Representative gated and non-gated auto-correlation data and corresponding fits for 80% STED power. (C) Resolution (and anomaly factor, inset) determined from the analysis of the experimental STED-FCS data of Att488-PE with gated (blue) and non-gated (red) modality. (D) Diameter (FWHM) of the effective observation spot as determined from gated STED-FCS analysis of Atto488-PE (red circles), TopFluor-PE (green squares) and Abberior STAR 512-PE (orange triangles) diffusing freely in the fluid DOPC SLB.

As highlighted before (Fig. 1), gated detection realises an optimised STED performance, since in this way detection of fluorescence signal from the focal centre is preferably detected over peripheral contributions. We tested this gated STED-FCS approach on the TopFluor, Atto488 and Abberior Star 512 labelled PE lipid analogues, once again diffusing in the DOPC SLB. Fig. 3A exemplifies the gated detection on experimental data from Atto488-PE, where due to the time-gated detection the immediate fluorescence signal over a time *T_g_* after the excitation pulse was excluded from the analysis, and only those photons lying within the grey shaded time window were selected for further calculation of FCS data. Note that we as well have selected a finite length of the gated detection window, whose end position was chosen to where the fluorescence signal reached background signal level, avoiding unnecessary detection of noise [56]. The positions of *T_g_* were specifically determined for each dye, starting with a rather large (strict) *T_g_*, and moving to lower times until reaching a sufficient signal-to-noise ratio in the FCS data. The optimal gate positions were 3.8–10.4 ns (relative to the excitation pulse) for TopFluor, and 2.4–6.4 ns for Atto488 and Abberior Star 512. Fig. 3B compares representative raw FCS data generated for the gated and un-gated case for Atto488-PE. Clearly, the transit time (equal to the decay time of the curves) was significantly reduced by the gating, exemplifying the reduction of the effective observation spot size [49]. This is further exemplified by plotting the diameter of the observation spot as a function of the STED power *P_STED_* (Fig. 3C). This diameter is given as the full-width-at-half-maximum (FWHM) of the Gaussian assumed intensity distribution of the emitted fluorescence profile, and it was for each *P_STED_* calculated from the transit time *τ_D_*,

$$\mathrm{FWHM}(P_{\mathrm{STED}})=\mathrm{FWHM}(P_{\mathrm{STED}}=0)\sqrt{\frac{\tau_{D}(P_{\mathrm{STED}})}{\tau_{D}(P_{\mathrm{STED}}=0)}}$$

where *FWHM*(*P_STED_*) is the diameter of the observation spot at the STED power *P_STED_* and *FWHM*(*P_STED_* = 0) the diameter of the confocal spot, as determined from imaging fluorescent beads or from theory [36].

While we could observe diffusion in spots down to a FWHM of 40 nm when applying gating, this was only possible down to 90 nm without gating (Fig. 3C). Further, the inset in Fig. 3C shows the values of the anomaly factor α as a function of *P_STED_*, both with and without applying gating. In theory, no anomaly (i.e. *α* = 1) is expected for the diffusion of the PE lipid analogues in the DOPC SLBs, also for STED recordings [62]. However, without gating α deviated from this perfect value, reaching values down to 0.7 especially for large *P_STED_*. As pointed out before [49] (see Sections 3.2, 2.5) this is a consequence of assuming a spatial Gaussian emission intensity profile in FCS theory, which is practically not the case for large *P_STED_*, leading to bias in fitting and thus an *α* < 1. Yet, applying gating recaptures a Gaussian emission intensity profile [49], and thus an anomaly of 1 was retained. The size of the observation spots of down to 40 nm in diameter is in the range achieved for bright and photo-stable red-emitting dyes (such as Atto647N) in the all-pulsed STED modality, as used in previous live-cell investigations [15], [36], [42]. However, using gated STED and the green emitting labels we now achieved such small observation spots already at 50% STED power, i.e. for *P_STED_* = 106 mW of CW-STED light, which is approximately half of the time-averaged power applied in the all-pulsed experiments. Yet, we have to note that the signal-to-noise ratio of the gated STED experiments is conceptually lower than that of the all-pulsed implementation [49]. An accurate comparison of both modes (gated vs. all-pulsed) has to employ the same label on the same setup, i.e. instituting both a CW and pulsed STED laser source, which is currently not available. Yet, we anticipate such experiments in the future.

Of the three selected PE lipid analogues, we reached the smallest observation spots for Atto488-PE (FWHM = 42 ± 4 nm), almost similar for Abberior Star 512-PE (FWHM = 53 ± 1 nm), and only FWHM = 86 ± 8 nm for the TopFluor-PE (Fig. 3D). Diffusion of Abberior Star 512-PE could not be monitored at the highest possible STED powers due to its limited brightness (cpm, see Fig. 2B), prevailing the recording of accurate FCS data in even smaller observation spots.

### 3.3 STED-FCS: tuning of the observation spot by gating

As highlighted previously [49], [56], the size of the observation spot can be tuned both by the STED power *P_STED_* as well as by the position of the time gate *T_g_*. A strict time gate, i.e. a large *T_g_*, rejects a large amount of signal and gives a more confined effective observation spot. The spot size increases as less and less signal is suppressed and *T_g_* is lowered, i.e. moved more towards the excitation pulse. This opens up the possibility to create FCS data for different observation spot sizes out of a single measurement at a single STED power [49]. However, for small *T_g_* the fluorescence emission intensity profile is not Gaussian anymore, introducing the aforementioned biased anomaly in the FCS analysis (compare Section 3.2 and inset Fig. 3C) [49]. A remedy to this is to limit the gating to small detection windows, especially for small *T_g_*, since contributions from a large range of STED laser actions are then avoided [63].

**Fig. 4:**
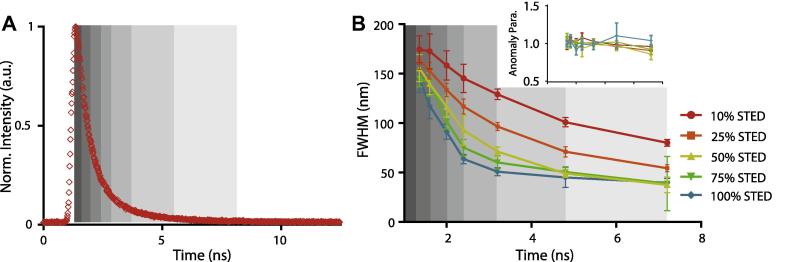
Tuning resolution by gating as exemplified for Atto488-PE diffusing freely in the DOPC SLB. (A) Fluorescence decay (red) and positions of the gates (differently grey shaded areas: 1.12–1.36, 1.36–1.6, 1.6–2.0, 2.0–2.4, 2.4–3.2, 3.2–4.8, 4.8–7.2 ns). (B) Diameter (FWHM) of the effective observation spots obtained with varying STED power and time-gates shown in (A), as determined from the experimental STED-FCS data.

Fig. 4B shows the diameter FWHM of the effective observation spot extracted from FCS recordings of Atto488-PE diffusing in the DOPC SLB for different gating conditions. The gating positions differed in both the start *T_g_* and width of the detection window, as highlighted by the grey shaded areas in Fig. 4A. For each STED power, the dependency of FWHM on the gating position (given as the starting point *T_g_* and the detection window as grey shaded area in Fig. 4B) was extracted from a single measurements, and error bars resulted from the dependencies generated from five independent measurements (Fig. 4B). The data clearly shows that one can extract parameters from FCS data over a large range of observation spot sizes out of a single measurement at a single STED power. The dynamic range in FWHM was 80–>180 nm for 10% STED power (21 mW), and 40–150 nm for >50% STED power. Note, that we increased the width of the gating windows for larger starting points *T_g_*. This is because of the lower fluorescence intensity at longer times, and a larger collection window ensures the collection of sufficient photons for reliable analysis. Being able to extract FCS data for multiple observation spot sizes and thus the dependency of the transit time on the observation spot diameter out of one single measurement enhances the strength of the gated STED-FCS technology even further. Moreover, it is worth noting that the maximum resolution that can be achieved by using maximum STED power can now also be reached by applying time gating at a lower *P_STED_* (Fig. 4B). This will allow observing the changes of the diffusion modes of molecules over time, with a time resolution of the acquisition time of a single FCS curve (i.e. usually 10 s), with less STED power, and all in a straightforward way on a commercial microscope with commercially available probes. However, again we have to note that the improved confinement of the observation spot by this gating modes comes at the expense of a decreased signal-to-noise ratio, demanding e.g. for longer measurement times.

## 4 STED-FCS pitfalls

It is inevitable that efficient inhibition of the fluorescence emission in the periphery of the confocal observation spot requires that quite large intensities of the added STED laser are employed. This is because, the stimulated emission process is competing against the usually <4 ns (lifetime) fast spontaneous fluorescence decay. It is however important to keep in mind that the peak intensities of the STED laser are in the range of those used for multi-photon microscopy. Nevertheless, great care has to be taken that the STED laser light does not introduce any unwanted effects such as photo-bleaching of the fluorescent labels, direct excitation of fluorescence emission, photo-toxic effects such as light-induced changes of the plasma membrane or cell death, heating, or optical trapping. Conceptually, the STED light is not absorbed by the label and does not produce any photo-reactive and thus photo-toxic species, unlike in multi-photon microscopy [64]. Unfortunately, excited state absorption of STED light can cause severe photo-bleaching [65]. Therefore, several control experiments have been reported for STED-FCS experiments. For example, it could be shown that – as expected from theory – the decrease of the average transit time *τ_D_* with increasing STED intensity coincided well with the decrease of the average number of fluorescent molecules in the observation area, a correlation that could only be explained by the optically controlled decrease of the observation spot’s length scale using stimulated emission [15]. Further, scanning of the beam during recording reduces the time the beam continuously spends on a certain spot, thereby minimizing aforementioned light-induced stress [66], [67], [68]. Yet, scanning of the beam during STED-FCS recordings realised the same results as of the single-point STED-FCS experiments [15], [41]. In addition, STED-FCS measurements on fluid model membranes did not reveal any sign of optical trapping [62]. In the meantime, parts of the STED-FCS experiments could be confirmed by fast single-molecule tracking experiments [69], [70], as well as near-field microscopy observations [27] using much lower laser intensities.

Intuitively, the use of a relatively large organic dye label will influence the dynamics of the lipids. While one never can fully exclude such bias, extensive control experiments indicated that, apart from a label-induced change in the lipids’ preference for more molecular ordered environments [41], [42], [62], [71], the molecular interaction and diffusion dynamics of the fluorescent lipid analogues hardly depended on the properties and position of the dye label but rather on the chemical structure of the lipid [15], [37], [42]. Only the introduction of a very polar dye by acyl-chain replacement introduced biased diffusion, namely faster mobility and negligible trapping (most probably from the polar label avoiding the hydrophobic membrane environment) [15], [71]. Further controls could rule out improper or unspecific incorporation of the lipid analogues into the cellular plasma membrane [15], [37].

## 5 STED-FCS variations and future developments

The STED-FCS technology has proven itself as a minimal invasive method to measure the dynamics of fluorescent lipid analogues in model and cell membranes in a direct manner. The technology has recently seen a number of variations allowing for further access to the spatial and temporal heterogeneity of molecular diffusion. In this way, STED-FCS data acquisition has been combined with fast beam-scanning of the lasers realising scanning STED-FCS [40], [41], [72], STED Raster Image Correlation Spectroscopy (RICS) [73], and STED cross-pair-correlation function analysis STED(pCF) [39], [74]. Scanning of the beam during STED-FCS recordings bears another advantage, since image correlation analysis can be employed to determine the size of the observation spot, making additional calibration measurements obsolete [39], [40], [41], [72]. Current and future developments will likely see the combination of STED with other fluorescence spectroscopy tools such as with fluorescence intensity distribution analysis (FIDA) or number and brightness (N&B) analysis [75], [76], [77]. In addition, complementary methods to STED-FCS or svFCS will arise, such as recent image mean square displacement (iMSD) analysis [9], which all in one way or the other allow access to the dependency of an apparent diffusion coefficient on observation spot sizes. The implementation of STED-FCS on commercial available microscopes will extend its use to a broader circle of users, not limiting the method to specialised optical laboratories.

A technological advancement that is still missing for gaining further insights into the molecular interactions so far studied with STED-FCS is the extension to the simultaneous detection of several labels. In analogy to conventional fluorescence cross-correlation spectroscopy (FCCS) [78], correlating the STED recordings of two differently labelled molecules will disclose their potential binding kinetics. Similar to simultaneous multi-colour STED microscopy [79], [80], the most promising approach will be the implementation of two fluorescent labels with distinct excitation wavelengths but a common STED laser.

Similar to the two-dimensional diffusion in a membrane, a shortening of the average transit time can in principle equally well be observed for 3-dimensional diffusion when moving from diffraction-limited confocal to 3-dimensional STED recordings. While this makes STED-FCS measurements in solution or inside the cellular cytosol feasible, these measurements are challenged by a lowered signal-to-background ratio due to non-inhibited out-of-focus fluorescence signal [35], [36].

Advances in labelling technology have significantly contributed to the development of STED microscopy [31], [32], [33], [44]. For the STED-FCS experiments on lipid membrane dynamics, it will be very important to study a functional fluorescent lipid analogue that does not alter the lipid’s preference for more ordered environments and is compatible with STED-FCS, such as introduced recently [41], [62]. Such a probe should be able to access areas of high molecular order, and will therefore be an important complement to investigate coalescence of potential signalling forms and existence of lipid nanodomains (or “rafts”) in the plasma membrane of living cells. Experiments using fluorescent lipid analogues with preferences for different molecular ordered environments revealed no correlation between this characteristic and anomalous diffusion in living cells so far [41], [71]. It will therefore be very important to investigate lipid membrane dynamics after triggering of cellular functions including activation of different receptors [1].

## 6 Conclusions

STED-FCS is a sensitive and unique tool for studying nano-scale membrane organisation, and determining the cellular functions and molecular interdependencies of membrane components. In this report, fluorescently-labelled lipids were investigated, however, the technique can be applied to membrane proteins as well [15], [72], [81]. More generally, STED-FCS expands currently available optical microscopy and spectroscopy techniques to the nanoscale and opens up exceptional possibilities to characterise and disclose complex cellular signalling events and therefore new approaches for drug screening and development [82]. Here we have shown a straightforward access to this technology using a commercially available turnkey microscope that does not require any modifications. Especially, we have identified Atto488 as an organic dye that performs well for STED-FCS studies as it shows high photo-stability, high molecular brightness, and is well depleted by the 592 nm STED laser to give a high spatial resolution of down to 40 nm in a time-gated approach. Further, we have demonstrated that the time-gated STED-FCS approach allows for creating FCS data for different observation spots from a single measurement, significantly simplifying the recording of STED-FCS data.
